# Abundance, Diversity and Distribution of Mosquito Species and Molecular Detection of Its Associated Hepatitis C Virus in Sharkia Governorate, Egypt

**DOI:** 10.3390/insects16040433

**Published:** 2025-04-19

**Authors:** Tharwat A. Selim, Sanad H. Ragab, Saber A. Riad, Randa I. Eltaly, Shaimaa H. Mohammed, Somia Eissa Sharawi, Naser Ahmed Alkenani, Ruoa Saleh Almahallawi, Hayat S. Al-Rashidi, Mohamed A. M. El-Tabakh

**Affiliations:** 1 Department of Zoology and Entomology, Faculty of Science (Boys), Al-Azhar University, Nasr City 11884, Cairo, Egypt; sanadragab@azhar.edu.eg (S.H.R.); saberiad60@azhar.edu.eg (S.A.R.); dr.m.eltabakh.201@azhar.edu.eg (M.A.M.E.-T.); 2 Department of Zoology and Entomology, Faculty of Science (Girls), Al-Azhar University, Nasr City 11884, Cairo, Egypt; randa.eltaly@azhar.edu.eg (R.I.E.); shimaamohamed.sci.g@azhar.edu.eg (S.H.M.); 3 Department of Biological Sciences, Faculty of Science, King Abdulaziz University, Jeddah 21589, Saudi Arabia; sesharawi@kau.edu.sa (S.E.S.); nalkenani@kau.edu.sa (N.A.A.); 4 Department of Biology, University College of Duba, University of Tabuk, Tabuk 71491, Saudi Arabia; ralmahlawi@ut.edu.sa; 5 Department of Biology, College of Science, Qassim University, Buraydah 52571, Saudi Arabia; h.alreshidi@qu.edu.sa

**Keywords:** mosquitoes, abundance, species composition, distribution, HCV prevalence, Sharkia Governorate

## Abstract

The presence of mosquitoes in a certain area is sufficient evidence of the transmission of diseases, in addition to the inconvenience and harassment they cause to the population. In order for mosquito control to be successful, there must be sufficient reports and information about the extent of the distribution and spread of the mosquito in this area. This study was conducted seasonally to calculate the distribution and abundance of mosquitoes and to initially detect hepatitis C virus in the most abundant species collected in some localities: Kafr Saqr (Hanot, El-Kodah, Abo Shokok, El-Hagarsa); Abo Kebeer (Manshat Radwan, Bane Aiat, Al-Rahmania, Horbat); Diarb Negm (Safor, El-Asaied, Karmot Sahbara, Saft Zreka); and El-Zakazik (Om El-Zein, Bany Amer, Al-Zankalon, Shanbrt El-Mimona) along Sharkia Governorate. According to the results, 12 mosquito species belonging to two genera were recorded in four stations (16 sites). The species *Culex pipiens* was found to be the most abundant across the majority of locations. It is reaching its peak in Al-Zankalon with a maximum count of 139 ± 7.61 individuals. *C. antennatus* exhibited a significant level of abundance (*p* < 0.001), especially in Al-Rahmania (82.5 ± 4.3). Species such as *C. perexiguus* and *An. pharoensis* were found in moderate-to-low numbers. The prevailing species is *C. pipiens* in all locations and seasons, especially during the summer. Other species like *C. antennatus* and *An. pharoensis* are also important, although their significance is somewhat less pronounced. Clusters of sites with similar features indicate that specific locations or species exhibit consistent patterns of activity or abundance throughout several seasons. These clusters represent groups of locations that share similar characteristics, as determined by the principal components. The findings of detecting hepatitis C virus in *C. pipiens* (the most abundant species) collected samples showed negative results for the presence of HCV during the summer season in Sharkia Governorate.

## Simple Summary

There is no doubt that mosquitoes represent the biggest problem in the world in terms of transmitting many pathogens. In order to solve this problem in a country, accurate information must be available about the distribution and bioecology of mosquitoes in that country. From this standpoint, the appropriate method and time can be chosen to control mosquitoes in the country. We conducted this entomological and molecular study in Sharkia Governorate during four seasons in order to better know the diversity and the distribution of mosquitoes and to detect the hepatitis C virus in the most common species.

## 1. Introduction

The special geographic location of Egypt aggravates the complexity and difficulty of mosquito-borne virus (MBV) surveillance and control [1]. Several researches in the field of mosquito control to reduce the spread of diseases [2,3,4,5], but it still poses a threat of transmitting many pathogens [6,7,8]. To develop an integrated mosquito control program in a certain area, we must have sufficient information about the distribution and spread of mosquitoes in that area [9]. Accordingly, the appropriate method of control can be chosen, thus reducing the spread of viruses and diseases in this area. At least five MBVs have been recorded in Egypt: dengue virus (DENV), Rift Valley fever virus (RVFV), Sindbis virus (SINV), West Nile virus (WNV), and Chikungunya virus (CHIKV) [10]. Hepatitis C virus (HCV) is one of the viruses that differ in the way it is transmitted by mosquitoes until it was proven beyond doubt that HCV is not transmitted by mosquitoes biologically, but it can be transmitted mechanically through the mouth parts of the mosquito [6,7,8,11]. HCV is an enveloped positive-strand RNA virus that belongs to the family Flaviviridae [12]. HCV is the causative agent of the majority of cases of transfusion-associated and community-acquired Non-A and Non-B hepatitis worldwide. Every year, about 339,000 deaths can be attributed to complications caused by HCV infection [13]. A basic attribute of mosquito populations is abundance; consequently, its estimation is one of the most important activities of mosquito ecologists. Abundance is a key factor in various types of studies, including life table analyses, assessment of control strategies, and estimation of vectorial capacity [14,15]. Furthermore, mosquito vectors often shift their feeding preference seasonally or spatially, depending on the availability of the blood meal source [14,15]. However, transmission of viruses across international borders can be attributed to the import and export of livestock and related products, as well as the translocation of mosquitoes and human travel and transportation [16]. Potential arthropod vector identification is important to understand which mosquito species may maintain the virus during inter-epidemic phases. It is also important to gain a clear understanding of the community composition and abundance of such mosquito species that may act as a vector for viruses survival and transmission [17,18]. A proper understanding of the relationship between agricultural activity and the occurrence, abundance and distribution of mosquito densities may provide information relevant to the development and implementation of an Integrated Vector Management (IVM) program based on adult productivity and variability. The aim of the current study is to know the spatial and temporal distribution of mosquito species in El Sharkia Governorate, Egypt, and to identify the most abundant species. In addition, the study aimed to detect the hepatitis C virus associated with abundant species based on the possibility of its mechanical transmission.

## 2. Materials and Methods

### 2.1. Study Area

**Figure 1:**
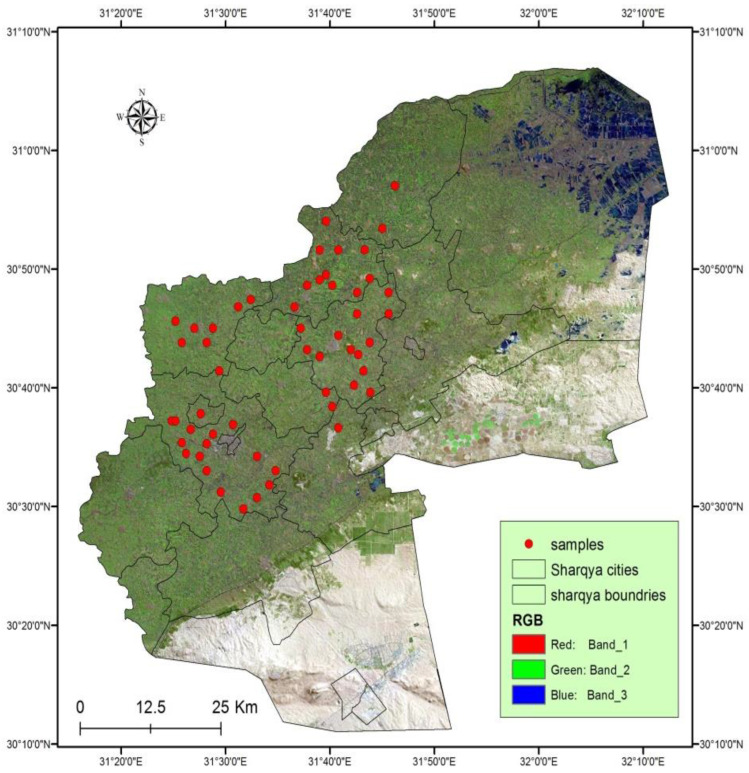
Map showing the location of sampling sites in Sharqia governorate in Egypt (map source: IESR, GIS 113unit, and Google map).

The El Sharqia Governorate is located in the northern Nile Delta of Egypt, spanning approximately between longitudes 31°15ʹ and 32°15ʹ E and latitudes 30°20ʹ and 31° N. With a territory of 4922 km^2^, it surpasses the Al-Behaira Governorate to become the second-largest governorate in the Nile Delta region. Four stations were chosen to cover most of the governorate. Each station was divided into four sites, and each site contained four points from which species were collected as follows: (A. Kafr Saqr (Hanot, El-Kodah, Abo Shokok, El-Hagarsa); B. Abo Kebeer (Manshat Radwan, Bane Aiat, Al-Rahmania, Horbat); C. Diarb Negm (Safor, El-Asaied, Karmot Sahbara, Saft Zreka); D. El-Zakazik (Om El-Zein, Bany Amer, Al-Zankalon, Shanbrt El-Mimona)) (Figure 1).

### 2.2. Field Trips and Sample Collection

Mosquitoes were surveyed in several field trips for one year from summer, July 2021 to spring, March 2022, in different selected stations along the Sharqia Governorate. The CDC light trap was used in our study. The baits used in this trap were carbon dioxide. One trap was placed at each study point for 24 h collection of mosquitoes. For the day collection traps were set around 10.00 a.m.–10.30 a.m. and collected on the second day at the same time. Four replicates were conducted at each study site.

### 2.3. Identification of Species

Collected traps were sealed into a plastic bag to be identified morphologically under a microscope (**Brand:** LEICA, **Model:** ATC 2000 Binocular) in the laboratory using identification keys as described by [19,20,21].

### 2.4. Real-Time PCR Analysis

The most abundant collected mosquitoes (*C. pipiens*) species were placed in a small amount of normal saline solution under freezing conditions to detect HCV [11] as follows. Total RNA was extracted using the TRIzol™ reagent (Invitrogen, Thermo Fisher Scientific, Waltham, MA, USA) according to the manufacturer’s protocol 12 12, followed by quantification and quality assessment. The extracted RNA was then used for real-time PCR to detect HCV 11 11 as the following. ABI prism 7300 was used as equipment that quantified the titer of the virus; this technique depends upon one-step detection of HCV, and the procedures were performed with the aid of the genesig^®^ standard kit for Hepatitis C Virus by Primerdesign™ Ltd. (Eastleigh, UK). All estimations were conducted in correspondence with positive and negative controls.

### 2.5. Working Protocol

Resuspend all components of the kit in DNA/RNA-free water according to the manual and spin thoroughly. Extract total RNA using the TRIzol™ reagent (Invitrogen, Thermo Fisher Scientific, Waltham, MA, USA) following the manufacturer’s instructions. Assess RNA quality and quantity before proceeding. Prepare a standard curve of HCV using different concentrations of the positive HCV sample. Amplification conditions using oasig OneStep2X RT-qPCR master mix according to Table 1.

### 2.6. Data Analysis

Data were coded and entered using the statistical package SPSS V. 22. Data was tested for satisfying assumptions of parametric tests, and continuous variables were subjected to the Shapiro–Wilk and Kolmogorov–Smirnov tests for normality. Probability and percentile data were standardized for normality using Arcsine Square Root. Data were presented as median and standard deviation, with analysis based on three replicate traps at each site across four sites in each geographical area; post-hoc analysis was evaluated using the Tukey pairwise comparison; *p* value was considered significant at <0.05. The analysis was evaluated using MiniTab V 14. Data were visualized when possible, using R studio V 2022.02.4.

## 3. Results

### 3.1. The Spatial Distribution of Mosquito Populations

An assessment was conducted to determine the spatial distribution of mosquito populations in several locations (Kafr Saqr, Abo Kebeer, Diarb Negm, El-Zakazik) across the four seasons (summer, autumn, winter, and spring). The following mosquito species were taxonomically classified: *C. perexiguus*, *Culex deserticola*, *Culiseta sp.*, *C. antennatus*, *C. pipiens*, *C. univittatus*, *An. anopheles*, *An. sergenti*, *An. multicolor*, *An. coustani*, *An. tenebrosus* and *An. pharoensis*. During the summer season, the species *C. pipiens* was found to be the most abundant across the majority of locations, reaching its peak in Al-Zankalon with a maximum count of 139 ± 7.61 individuals. *C. antennatus* exhibited a significant level of abundance (*p* < 0.001), especially in Al-Rahmania (82.5 ± 4.3). Species such as *C. perexiguus* and *An. pharoensis* were found in moderate to low numbers. The analysis of variance (ANOVA) results for the summer season showed statistically significant variations in the number of mosquitoes between the different sites and species (*p* < 0.001 for both). This suggests that there is significant spatial variability and distinct distribution patterns for each mosquito species. Tukey pairwise comparisons revealed that Shanbrt El-Mimona had significantly greater mosquito abundance in contrast to many other sites (e.g., Abo Shokok, *p* < 0.001).

**Figure 2:**
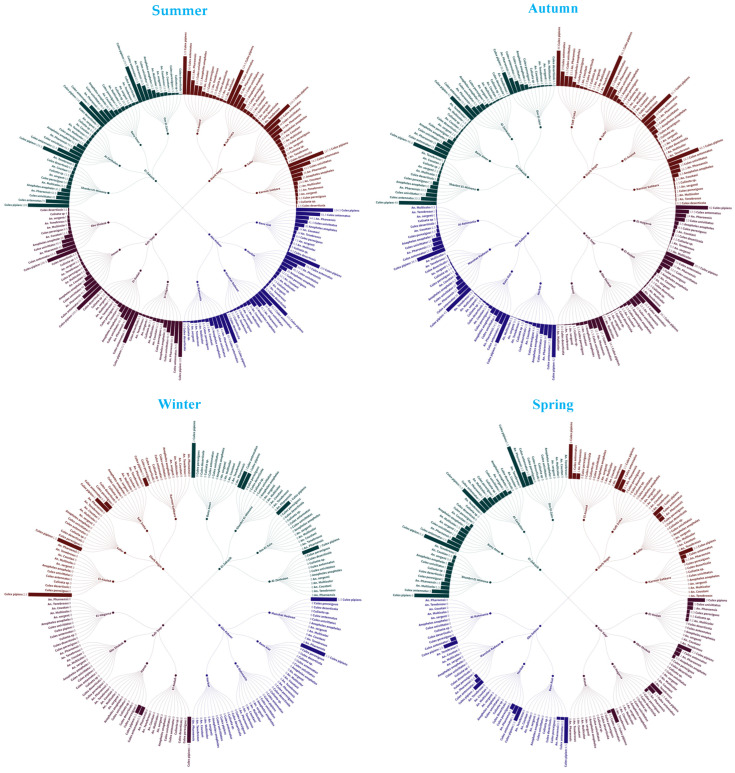
Radial bar tree represents mosquito abundance over different seasons.

Through autumn, there was a significant decrease in the number of mosquitoes for most species in comparison to the summer (*p* < 0.001). The population of *C. pipiens* was consistently high, reaching its peak at El-Asaied with a value of 56.5 ± 1.75. A significant decline (*p* < 0.01) was seen in other species, such as *C. perexiguus* and *Culex deserticola*. The ANOVA findings showed a substantial variance attributed to species (*p* < 0.001), but the differences between sites were not statistically significant (*p* = 0.079). Tukey comparisons revealed no statistically significant variations among sites (*p* > 0.05), indicating a more uniform distribution across locales during the autumn season (Table 1 and Figure 2).

In winter, there were very few mosquitoes of any species, with only rare sightings of *C. pipiens* in Saft Zreka (2.5 ± 0.28). The ANOVA analysis revealed significant variations attributed to species and the interactions between sites and species (*p* < 0.001). However, the differences between sites alone were not found to be significant (*p* = 0.073). This suggests that although there was a low overall abundance, the limited instances were affected by both the location and the type of species. During the spring season, there was a significant increase in the number of mosquitoes, specifically *C. pipiens*, in various locations, with Shanbrt El-Mimona (1.25 ± 0.158) being particularly notable. *C. antennatus* exhibited heightened activity at El-Asaied, with an average of 1.5 ± 0.64. The ANOVA analysis conducted on the spring data revealed statistically significant differences among the various sites, species, and their interactions (*p* < 0.001). Tukey pairwise comparisons indicated substantial variations across the sites, with Shanbrt El-Mimona exhibiting much greater abundance compared to several other sites (e.g., Al-Rahmania, *p* < 0.001) (Table 1 and Figure 2).

### 3.2. The Spatial and Temporal Patterns of Mosquito Populations

**Figure 3:**
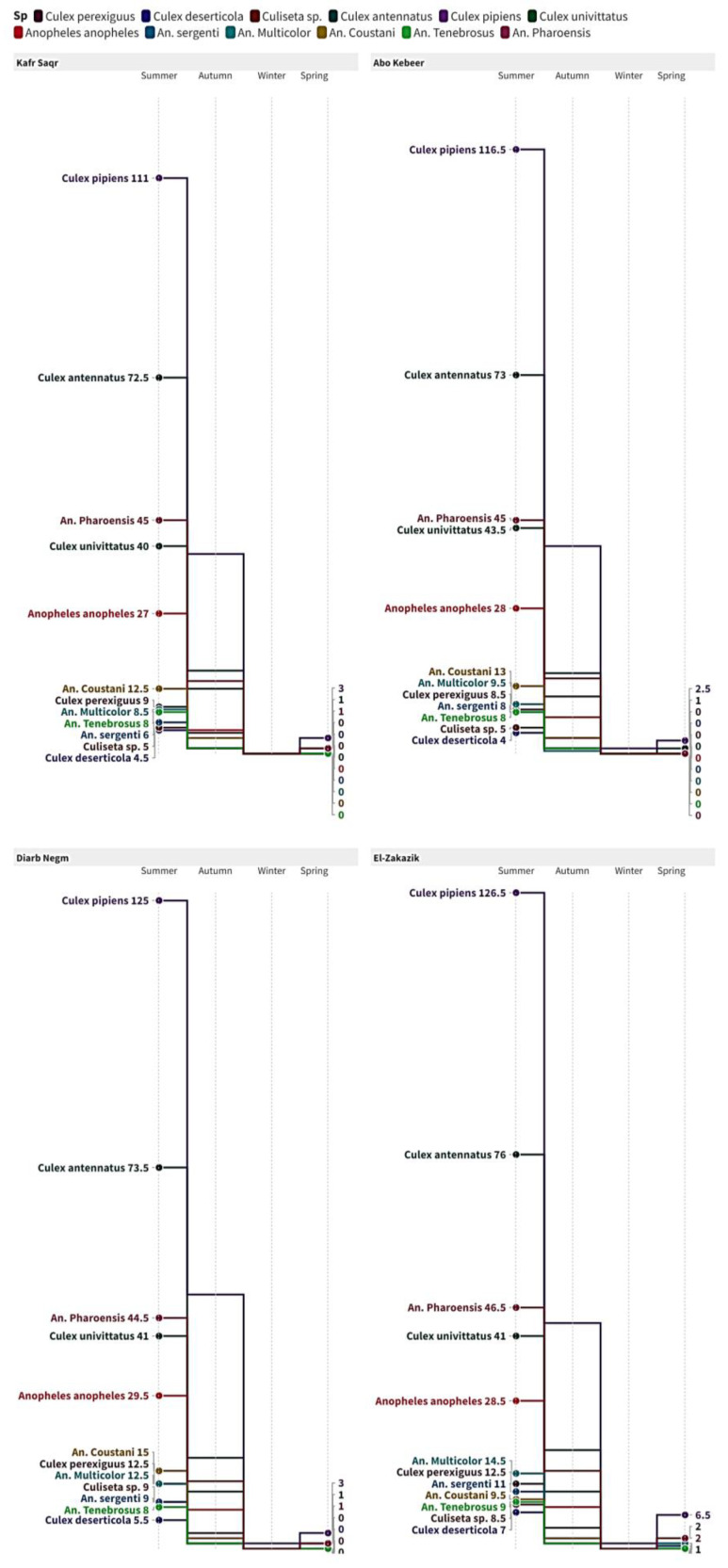
Step-slope chart.

The spatial and temporal patterns of mosquito populations are greatly influenced by both the specific location and the species of mosquitoes. The notable site and species interactions during the summer and spring emphasize the significance of focused vector control tactics, taking into account the varied distribution of mosquito species. The step-slope chart (Figure 3) provides a thorough depiction of the distribution and prevalence of different mosquito species across various areas and seasons. The prevailing species is *C. pipiens* in all locations and seasons, especially during the summer. Other species like *C. antennatus* and *An. pharoensis* are also important, although their significance is somewhat less pronounced. The map accurately depicts the seasonal fluctuations and the comparative prevalence of observed mosquito species, offering significant insights into their geographical distribution across the four regions.

**Figure 4:**
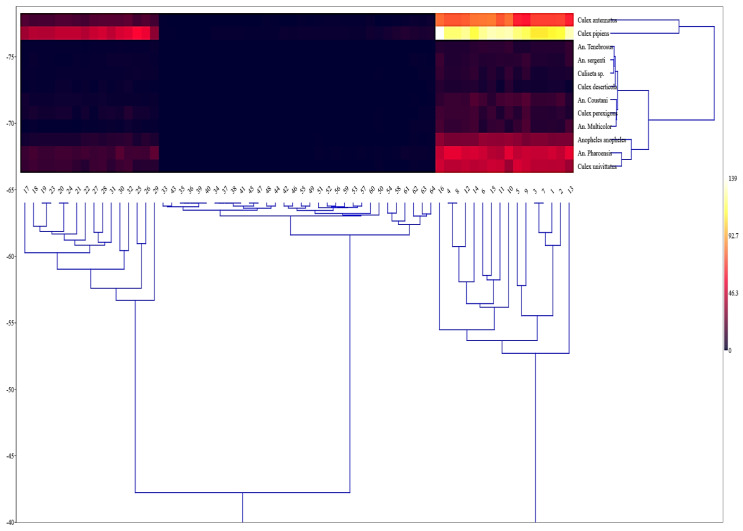
Two-way heat map.

The heatmap (Figure 4) depicts the spatial and temporal distribution of diverse locations, revealing distinct patterns in the data. The dendrograms displayed at the top and left of the heatmap depict the hierarchical grouping of sites, providing insights into the linkages and similarities in their mosquito abundance. Clusters of sites with similar features indicate that specific locations or species exhibit consistent patterns of activity or abundance throughout several seasons. The presence of distinct clusters suggests that some locations exhibit elevated abundance under specific circumstances, which are depicted by lighter hues ranging from yellow to red. Conversely, lower values are visualized by darker shades, ranging from black to dark red. This pattern indicates that certain species exhibit higher levels of activity or population density in distinct geographical regions and time periods. The heatmap provides a clear and informative visualization of the hierarchical relationships and seasonal patterns of distinct locations. It also offers useful insights into the distribution and behavior of species across various regions and throughout different times of the year.

**Figure 5:**
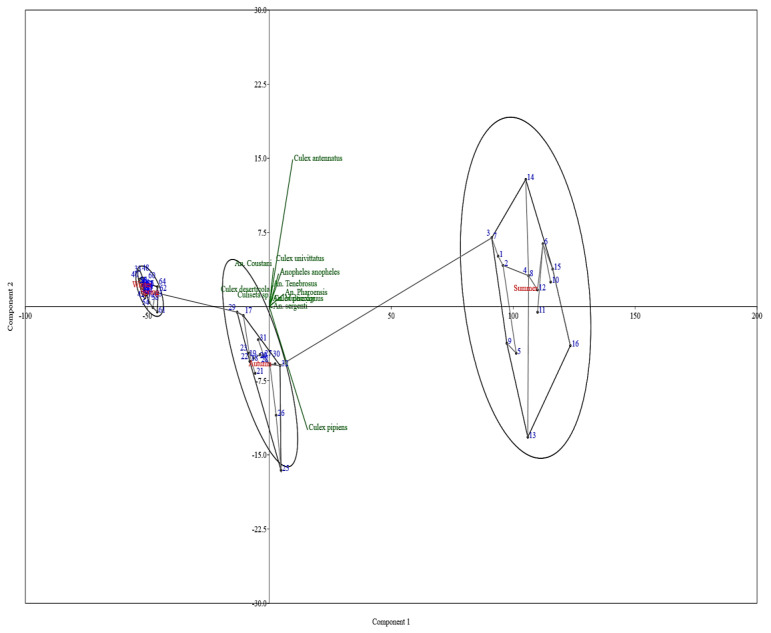
PCA.

The PCA plot (Figure 5) efficiently demonstrates the distribution and clustering of different sites and species by utilizing their principal components. This provides a clear depiction of the main patterns and variations present in the present dataset. The fundamental sources of variance are shown by the two principal components on the *X* and *Y* axes. The relative similarities and differences between sites and species can be determined by their positioning on this plot. Three separate groups of sites are clearly visible, each contained within an ellipse (the first for summer, the second for autumn and the third for winter and spring together). These clusters represent groups of locations that share similar characteristics, as determined by the principal components. Winter–spring clusters exhibit a concentrated aggregation of sites with minimal deviation along both components, suggesting uniform conditions or species distributions within these sites. The summer cluster exhibits a wider distribution along component 1, indicating a diversity in species and environmental circumstances that are represented by this component. In contrast, the autumn cluster is more strict to component 2, with much less deviation than the summer cluster.

The chart illustrates the distribution of various sites across different areas and seasons. In the summer, sites in Kafr Saqr include Hanot (Code 1), El-Kodah (Code 2), Abo Shokok (Code 3), and El-Hagarsa (Code 4). Abo Kebeer features Manshat Radwan (Code 5), Bane Aiat (Code 6), Al-Rahmania (Code 7), and Horbat (Code 8). Diarb Negm’s sites are Safor (Code 9), El-Asaied (Code 10), Karmot Sahbara (Code 11), and Saft Zreka (Code 12). El-Zakazik includes Om El-Zein (Code 13), Bany Amer (Code 14), Al-Zankalon (Code 15), and Shanbrt El-Mimona (Code 16). In autumn, Kafr Saqr sites are Hanot (Code 17), El-Kodah (Code 18), Abo Shokok (Code 19), and El-Hagarsa (Code 20). Abo Kebeer features Manshat Radwan (Code 21), Bane Aiat (Code 22), Al-Rahmania (Code 23), and Horbat (Code 24). Diarb Negm’s sites are Safor (Code 25), El-Asaied (Code 26), Karmot Sahbara (Code 27), and Saft Zreka (Code 28). El-Zakazik includes Om El-Zein (Code 29), Bany Amer (Code 30), Al-Zankalon (Code 31), and Shanbrt El-Mimona (Code 32). In winter, Kafr Saqr sites are Hanot (Code 33), El-Kodah (Code 34), Abo Shokok (Code 35), and El-Hagarsa (Code 36). Abo Kebeer features Manshat Radwan (Code 37), Bane Aiat (Code 38), Al-Rahmania (Code 39), and Horbat (Code 40). Diarb Negm’s sites are Safor (Code 41), El-Asaied (Code 42), Karmot Sahbara (Code 43), and Saft Zreka (Code 44). El-Zakazik includes Om El-Zein (Code 45), Bany Amer (Code 46), Al-Zankalon (Code 47), and Shanbrt El-Mimona (Code 48). In spring, Kafr Saqr sites are Hanot (Code 49), El-Kodah (Code 50), Abo Shokok (Code 51), and El-Hagarsa (Code 52). Abo Kebeer features Manshat Radwan (Code 53), Bane Aiat (Code 54), Al-Rahmania (Code 55), and Horbat (Code 56). Diarb Negm’s sites are Safor (Code 57), El-Asaied (Code 58), Karmot Sahbara (Code 59), and Saft Zreka (Code 60). El-Zakazik includes Om El-Zein (Code 61), Bany Amer (Code 62), Al-Zankalon (Code 63), and Shanbrt El-Mimona (Code 64).

### 3.3. Environmental Indices

**Figure 6:**
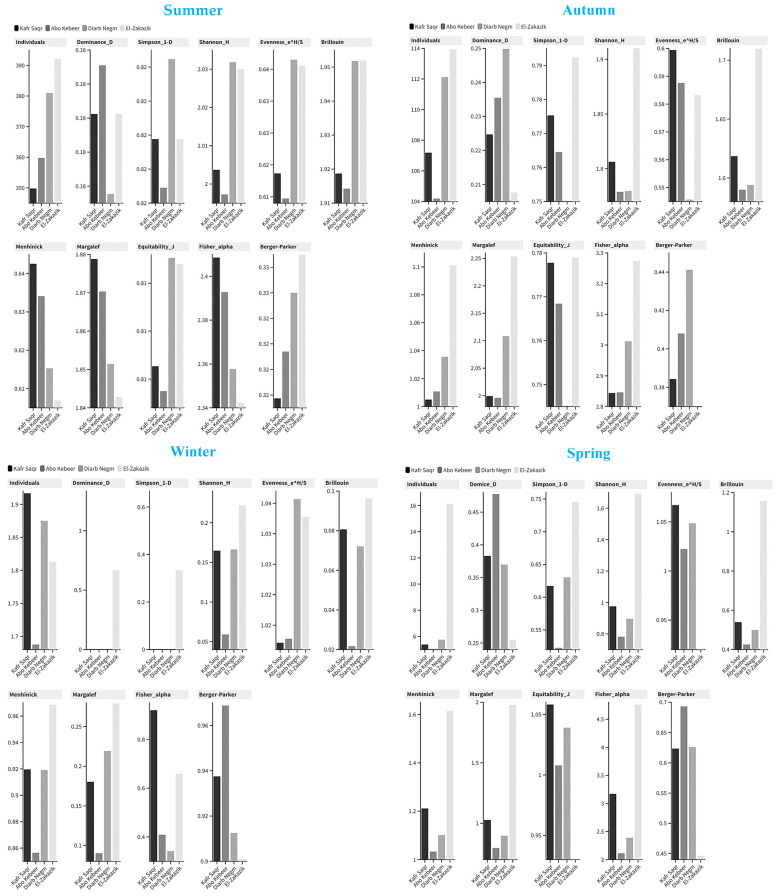
Gradient column chart.

During the summer season, El-Zakazik has the largest population density of individuals (392.06 ± 10.838), which is significantly more than that of other sites, as confirmed by the ANOVA analysis (*p* value < 0.01). The Dominance_D index exhibits consistency across all regions, with a value of approximately 0.18, suggesting a well-balanced distribution of dominating species. The Simpson_1-D index, a measure of variety, is somewhat greater in Diarb Negm (0.82 ± 0.005) compared to other locations (0.81). However, the analysis of variance (ANOVA) reveals no statistically significant differences (*p* value = 0.881). The Shannon_H index, which measures species richness, consistently hovers around 2.03. Although ANOVA analysis shows slight variations with no statistical deviation (*p* value = 0.141), the data imply a high level of species richness in all regions. The evenness indices (Evenness_e^H/S) in Diarb Negm and El-Zakazik are higher at 0.63, indicating a consistent distribution of species. The patterns of the Brillouin index are comparable, suggesting a moderate level of variability. The Menhinick and Margalef indices indicate higher levels of species diversity in Kafr Saqr and Abo Kebeer, suggesting a little greater richness of species (Table 2 and Figure 6).

Through autumn, there is a drop in the number in all regions, with El-Zakazik once again having the highest recorded individual index of 113.93 ± 9.445. The level of Dominance_D exhibits a minor increase, particularly in Diarb Negm (0.24 ± 0.014), suggesting a greater dominance by a smaller number of species. The Simpson_1-D index reaches its peak in El-Zakazik (0.79 ± 0.004), indicating a higher level of diversity. This is further supported by the ANOVA analysis, which reveals significant differences (*p* value = 0.003). The Shannon_H index in El-Zakazik is much higher at 1.91 ± 0.024, as indicated by the significant ANOVA results (*p* value = 0.002). The level of evenness in Diarb Negm stays quite stable, although there is a minor drop. The Fisher_alpha index, which measures species diversity, is highest in El-Zakazik with a value of 3.27 ± 0.072. The ANOVA test reveals that there are significant regional differences, with a *p* value of 0.000. The Berger-Parker index indicates that Diarb Negm has the highest level of dominance, with a value of 0.44 ± 0.021 (Table 2 and Figure 6).

On the other hand, winter shows a substantial decrease in the population of individuals in all regions. This decline is characterized by extremely low values and a high degree of variability. The Dominance_D and Simpson_1-D indices cannot be estimated for certain regions due to a lack of sufficient observations. The Shannon_H, Evenness, and Brillouin indices exhibit a remarkably low value, suggesting a lack of diversity and uniformity among species. The ANOVA analysis reveals no statistically significant variations. The Menhinick and Margalef indexes both indicate a low level of species diversity. The Berger-Parker index exhibits elevated values, signifying a limited number of species that dominate throughout the winter season (Table 2 and Figure 6).

Finally, during the spring season, there is a noticeable increase in the individual indices, particularly in El-Zakazik, with a mean value of 16.12 ± 1.252. The Dominance_D index in El-Zakazik (0.25 ± 0.081) exhibits a drop, suggesting a reduction in the dominance of a small number of species. The Simpson_1-D index indicates a greater level of variety in El-Zakazik (0.74 ± 0.081), as evidenced by statistically significant ANOVA results (*p* value = 0.083). The Shannon_H index is highest in El-Zakazik (1.69 ± 0.279), indicating a greater number of species and confirmed by ANOVA to have significant differences (*p* value = 0.000). The evenness indices of El-Zakazik are rather consistent, but they are slightly lower. The Fisher_alpha index at El-Zakazik is significantly high at 4.75 ± 1.358, which suggests a considerable level of species variety. The Berger-Parker index exhibits a decline in El-Zakazik, signifying a reduction in the dominance of a small number of species. This finding is supported by the analysis of variance (ANOVA) with a significant *p* value of 0.005 (Table 2 and Figure 6). The same findings were confirmed for the 16 sites belonging to the four stations disscused in Table S2.

### 3.4. Detecting of HCV on Collecting Samples

The findings showed that Cx. pipiens was the most prevalent species in all sites, and by detecting HCV in Cx. pipiens samples, the results were found to be negative for HCV during the summer season in Sharkia Governorate.

## 4. Discussion

Insects are powerful and rapid adaptive organisms with high fecundity rates and short life cycles. Human interruption in agroecosystems and global climatic variations are disturbing the insect ecosystem. According to the present study conducted along the Sharkia Governorate, 12 mosquito species, namely, *Cx. pipiens*, *Cx. antennatus*, *C.x univittatus*, *Cx. perexiguus*, *Cx. deserticola*, *Culiseta sp. An. coustani*, *An. pharoensis*, *Ano. tenebrosus*, *An. anopheles*, *An. sergenti* and *An. multicolor* belonging to two genera were recorded in four stations (16 sites). The results are similar to those of [19,22,23]. The current study revealed that the species *Culex pipiens* was found to be the most abundant across the majority of locations; for example, in the summer, it reaches its peak in Al-Zankalon with a maximum count of 139 ± 7.61 individuals. *C. antennatus* exhibited a significant level of abundance (*p* < 0.001), especially in Al-Rahmania (82.5 ± 4.3). Species such as *C. perexiguus* and *An. pharoensis* were found in moderate to low numbers. According to the same context [9], the most abundant genus in the Fayum governorate was *Culex*, which had four species and accounted for 88.01% of all mosquitoes, while *Anopheles* had six species and represented 11.99% of all mosquitoes. The most abundant and dominant mosquito species was *C. pipiens,* which was followed by *C. antennatus* and *C. bivittatus.* The rarest mosquito species were *An. multicolor* and *An. tenebrosus.* Nine species from four genera were found in mosquitoes collected from the Beheira Governorate, according to another study [24]. *Cx pipiens* 36.57% (13830/37820), *Cx. antennatus* 18.83% (7121/37820), *Cx. theileri* 8.48% (3209/37820), *Cx. tritaeniorhynchus* 5.78% (2188/37820), *Cx. Longiareolata* 11.97% (4526/37820), *Ae. caspius* 7.57% (2863/37820), *Ae. detritus* 2.83% (1070/37820), *An. multicolor* 3.79% (1434/37820), and *An. sergentii* 4.18% (1579/37820). Additionally, [25] found that the most abundant species were *An. pharoensis*, *C. pipiens*, *C. antennatus*, *C. univittatus*, and *An. coustani*. In every kind of breeding habitat, *C. pipiens* and *Cx. antennatus* were discovered. According to Sharkia [26], rice fields were plentiful, and *Cx. pipiens* larvae accounted for 83.4% of all larvae collected during the year. The larvae also preferred wells and cesspits. Similarly, at Giza, it was discovered that *Cx. pipiens* was the most prevalent mosquito species, accounting for 97.95 percent of all species [27]. The larvae were found in all breeding locations, particularly in seepage, canals, drains, cesspits, and wells. Also, [28] found that *Cx. pipiens* was the most common mosquito species in Gharbia. While [29] in Mansoura discovered that *Cx. univittatus, Cx. antennatus,* and *Ae. caspius* were the most prevalent, *Cx. deserticola* was the least common, and neither *Cx. pipiens* was found in rice fields, sewage wells, or drains. *Cx. antennatus* is only found in rice fields, and only a small portion was in a canal of Mansoura center. In four Egyptian governorates, *Cx. pipiens* and *Cx. antennatus* were the predominant species, according to a three-year study [30].

The present study revealed that the prevailing species is *C. pipiens* in all locations and seasons, especially during the summer. Other species like *C. antennatus* and *An. pharoensis* are also important, although their significance is somewhat less pronounced. Clusters of sites with similar features indicate that specific locations or species exhibit consistent patterns of activity or abundance throughout several seasons. These clusters represent groups of locations that share similar characteristics, as determined by the principal components. In agreement with [9], the spatial distribution of mosquitoes recorded at the Wadi El Rayan protected area revealed that site 2 had the highest abundance, with an annual average of 149 ind./trap, or roughly 27.07 percent of the total abundance. Site 1 had the second-highest abundance, at 143.5 ind./trap, or 26.07 percent, and site 3 had the third-highest abundance, at 137.25 ind./trap, or 24.2%. At 120.75 (21.93 percent of the total abundance), site 4 had the lowest abundance. Similarly, [31] discovered that these species were only found in large numbers in the Nile Delta, and [32] discovered them in the vicinity of Cairo and Oases. Additionally, [33] discovered these species in large quantities along the coast. Also, [34] found them among the commonest five mosquitoes of the Nile Delta. Perhaps the discrepancy in the density comes from the fact that the species prefers to have shelters outside human habitations. The same observations were given by [29]. Also, *Culiseta* sp. was recorded in canals, drains and sewage wells and not detected in rice fields in different areas. This differs from that of [31], who found it throughout the Nile Valley, and [32], who found it in the Oases, but it agreed with [35,36], who found it in the Suez Canal zone, around Cairo, in Faiyum, in Northern Delta and in some Oases. Moreover, *Cx. deserticola* was found only in a few numbers in canals and sewage wells. The results agreed with those published by [29].

Environmental indices showed a significant variation in all seasons. For example, during the summer season, El-Zakazik has the largest population density of individuals (392.06 ± 10.838). The Dominance_D index exhibits consistency across all regions, with a value of approximately 0.18, suggesting a well-balanced distribution of dominating species. The Simpson_1-D index, a measure of variety, is somewhat greater in Diarb Negm (0.82 ± 0.005) compared to other locations (0.81). The Shannon_H index, which measures species richness, consistently hovers around 2.03. The evenness indices (Evenness_e^H/S) in Diarb Negm and El-Zakazik are higher at 0.63, indicating a consistent distribution of species. The patterns of the Brillouin index are comparable, suggesting a moderate level of variability. The Menhinick and Margalef indices indicate higher levels of species diversity in Kafr Saqr and Abo Kebeer, suggesting a little greater richness of species. Similarly, [9] found that site 3 had the highest species richness value (1.666) in the summer, followed by site 2 with 1.619 in the fall, and site 1 with the lowest value (0.5386) in the winter, followed by site 3 with 0.5581. Additionally, site 1 had the greatest Shannon index value (1.703) in the summer, followed by site 2 with 1.663, but site 1 had the lowest value (0.9954) in the winter, followed by site 3 with 1.027. In the same context, the highest Evenness values were 0.9351 and 0.9061 during winter at site 3 and site 1, respectively, but the lowest Evenness values were 0.5236 at site 1 during spring and 0.5679 at site 3 during spring. Concerning the Simpson index, its highest value was 0.7642 and 0.7576 during summer at site 2 and site 2, respectively. However, the lowest value of the Simpson index was 0.5261 at site 1 during spring, followed by 0.5281 at site 4 during winter. This is comparable to [25], who found that the three agroecosystems and Fowa city showed notable differences in mosquito abundance and species richness. The observed disparities in the richness of aquatic habitats between the three villages and Fowa city could be the cause of these variations. A wider variety of mosquito species were supported by the irrigated agroecosystem due to its more varied habitat types. Although they can use the same habitats as Anopheles mosquitoes, Culex mosquitoes can breed in a variety of settings [37]. Prior research has documented a significant correlation between mosquito species richness and habitat type variety [38,39,40].

Given the possibility of mosquitoes transmitting HCV [6,7,8,11], we conducted a PCR analysis to detect the virus in the most abundant samples at all sites during the summer season. Our results revealed that *Cx. pipiens* is the most abundant species on all sites, and by detecting HCV in *Cx. pipiens* samples, the findings were found to be negative for HCV during the summer season in Sharkia Governorate. In contrast, [41] reported that HCV is associated with three mosquito species (*Ae. aegypti* (L.), *Ae. albopictus* and *An. stephensi*). On the other hand, [42,43] reported similar observations in *Cx. quinquefasciatus* that were fed on HCV-positive blood and in those caught in HCV-positive patients’ homes. Recently, several flaviviruses have been sequenced, characterized and identified in arthropods. For instance, in the near future, the Korean Peninsula may become the perfect biological setting for the spread of mosquitoes as disease vectors due to the gradual change in the country’s temperature from temperate to subtropical [44]. The most prevalent mosquito species in our survey is *Cx. pipiens.* There is an urgent need to investigate the role of *Cx. pipiens* in flavivirus transmission because of the high feeding rates of mosquitoes on mammals, including humans [45]. Additionally, ongoing globalization and significant climate change are major contributors to the spread of diseases carried by mosquitoes in Egypt, making these insects dangerous to the local population [15,46,47]. In the same vein, [48] examined the occurrence of flaviviruses in mosquitoes and found that ISF CxFV was present in *Culex* species in the ROK’s Jeju province’s urban regions. Using RT-nPCR, about 21 of the 207 pools had positive results for CxFV. Only 10.15 percent of the samples were positive (21/207 pools). The few positive samples are comparable to those reported in Vietnam in 2005 (26/1122 pools, 2.3%) and Kenya in 2007–2012 (only 0.3% pools were positive for CxFV) [49,50]. Furthermore, a study showed the nested PCR assay’s sensitivity for flaviviral identification of samples with low viral loads [51]. The majority of *Cx. p*. pallens’s abundance was noted in Jeju’s Jungang-dong neighborhood, indicating the species’ prevalence in urban areas [52]. In agreement with our research, every vector sample gathered from the various regions of the Jazan area tested negative for RVFV [53]. About 1823 mosquitoes were gathered in pools of five to ten during an inter-epidemic time and examined for the presence of RVFV in an entomological study conducted in 2012 in Tanzania’s Ngorongoro region; no positive samples were found [54]. The question of where and how the virus is maintained is raised, even though this could be related to the RVFV’s low level of circulation or nonexistence in the study location. The common occurrence of *Cx. pipiens* species and other culicine mosquitoes in an area contribute to the risk of other mosquito-borne disease transmission in this area [55]. Virus transmission is based on abundance, susceptibility to infection, the ability to transmit viruses, and feeding behavior [56]. Planning mosquito control strategies and studying the epidemiology of diseases spread by mosquitoes depend heavily on an understanding of mosquito habitats [57]. Our results are also in full agreement with the October 9 event where the World Health Organization congratulated Egypt on its unprecedented progress towards eliminating hepatitis C, becoming the first country to reach the “gold level” on the path to hepatitis C elimination according to WHO criteria. According to the 2023 WHO Guidance for country validation of viral hepatitis elimination and path to elimination, countries can apply for full validation of gold, silver or bronze tiers on the path to elimination based on achieving relevant targets [58]. Egypt is the first country that applied for validation and achieved gold tier status on the path to elimination, meaning that it is well on its way towards reaching all elimination targets before 2030.

## 5. Conclusions

This study has reported the distribution, abundance and diversity of mosquitoes collected across four seasons within Sharkia Governorate. Overall, the prevailing species is *C. pipiens* in all locations and seasons, especially during the summer. Other species like *C. antennatus* and *An. pharoensis* are also important, although their significance is somewhat less pronounced. Clusters of sites with similar features indicate that specific locations or species exhibit consistent patterns of activity or abundance throughout several seasons. These clusters represent groups of locations that share similar characteristics, as determined by the principal components. Also, by detecting HCV in *Cx. pipiens* samples, the findings were found to be negative for HCV during the summer season in Sharkia Governorate. Given mosquito breeding sites, health authorities could use a generated map identifying high-risk locations to forecast epidemics or endemic outbreaks and recommend workable mosquito control strategies.

## Figures and Tables

**Table 1:** The components of PCR in the reaction tube.

Component	Volume (µL)
oasig OneStep2X RT-qPCR master mix	10
HCV primer/probe mix	1
RNA template	5
DNA/RNA-free water	4
Final volume	20

**Table 2:** Environmental indices.

		Kafr Saqr	Abo Kebeer	Diarb Negm	El-Zakazik
**Summer**	Individuals	349.75 ± 6.815	359.75 ± 8.913	380.93 ± 6.418	392.06 ± 10.838
	Dominance_D	0.18 ± 0.001	0.18 ± 0.002	0.17 ± 0.005	0.18 ± 0.006
	Simpson_1-D	0.81 ± 0.001	0.81 ± 0.002	0.82 ± 0.005	0.81 ± 0.006
	Shannon_H	2 ± 0.008	1.99 ± 0.012	2.03 ± 0.025	2.03 ± 0.026
	Evenness_e^H/S	0.61 ± 0.005	0.61 ± 0.007	0.63 ± 0.016	0.63 ± 0.016
	Brillouin	1.91 ± 0.009	1.91 ± 0.012	1.95 ± 0.025	1.95 ± 0.026
	Menhinick	0.64 ± 0.006	0.63 ± 0.007	0.61 ± 0.005	0.6 ± 0.008
	Margalef	1.87 ± 0.006	1.87 ± 0.007	1.85 ± 0.005	1.84 ± 0.008
	Equitability_J	0.8 ± 0.003	0.8 ± 0.005	0.81 ± 0.01	0.81 ± 0.01
	Fisher_alpha	2.4 ± 0.011	2.39 ± 0.014	2.35 ± 0.009	2.34 ± 0.015
	Berger-Parker	0.31 ± 0.002	0.32 ± 0.003	0.32 ± 0.005	0.32 ± 0.012
**Autumn**	Individuals	107.18 ± 4.358	104.18 ± 3.402	112.12 ± 2.175	113.93 ± 9.445
	Dominance_D	0.22 ± 0.002	0.23 ± 0.009	0.24 ± 0.014	0.2 ± 0.004
	Simpson_1-D	0.77 ± 0.002	0.76 ± 0.009	0.75 ± 0.014	0.79 ± 0.004
	Shannon_H	1.8 ± 0.006	1.77 ± 0.033	1.77 ± 0.03	1.91 ± 0.024
	Evenness_e^H/S	0.59 ± 0.018	0.58 ± 0.012	0.54 ± 0.013	0.58 ± 0.013
	Brillouin	1.61 ± 0.008	1.58 ± 0.028	1.59 ± 0.028	1.7 ± 0.023
	Menhinick	1 ± 0.028	1.01 ± 0.03	1.03 ± 0.02	1.1 ± 0.036
	Margalef	1.99 ± 0.07	1.99 ± 0.045	2.1 ± 0.032	2.25 ± 0.026
	Equitability_J	0.77 ± 0.01	0.76 ± 0.01	0.74 ± 0.01	0.77 ± 0.009
	Fisher_alpha	2.84 ± 0.118	2.84 ± 0.09	3.01 ± 0.064	3.27 ± 0.072
	Berger-Parker	0.38 ± 0.011	0.4 ± 0.012	0.44 ± 0.021	0.37 ± 0.008
**Winter**	Individuals	1.91 ± 0.315	1.68 ± 0.236	1.87 ± 0.484	1.81 ± 0.199
	Dominance_D	---	---	---	0.66 ± 0.152
	Simpson_1-D	---	---	---	0.33 ± 0.152
	Shannon_H	0.16 ± 0.103	0.05 ± 0.058	0.16 ± 0.056	0.22 ± 0.087
	Evenness_e^H/S	1.01 ± 0.017	1.01 ± 0.017	1.04 ± 0.018	1.03 ± 0.023
	Brillouin	0.08 ± 0.05	0.02 ± 0.021	0.07 ± 0.024	0.09 ± 0.037
	Menhinick	0.91 ± 0.078	0.85 ± 0.071	0.91 ± 0.078	0.96 ± 0.053
	Margalef	0.18 ± 0.115	0.09 ± 0.09	0.21 ± 0.087	0.27 ± 0.115
	Equitability_J	---	---	---	---
	Fisher_alpha	0.91 ± 0.431	0.4 ± 0.162	0.34 ± 0.164	0.65 ± 0.25
	Berger-Parker	0.93 ± 0.039	0.96 ± 0.031	0.91 ± 0.029	0.9 ± 0.041
**Spring**	Individuals	5.37 ± 0.88	5.04 ± 0.715	5.75 ± 0.94	16.12 ± 1.252
	Domice_D	0.38 ± 0.089	0.47 ± 0.076	0.36 ± 0.078	0.25 ± 0.081
	Simpson_1-D	0.61 ± 0.089	0.52 ± 0.076	0.63 ± 0.078	0.74 ± 0.081
	Shannon_H	0.97 ± 0.25	0.78 ± 0.108	0.89 ± 0.098	1.69 ± 0.279
	Evenness_e^H/S	1.06 ± 0.023	1.02 ± 0.053	1.04 ± 0.051	0.92 ± 0.039
	Brillouin	0.53 ± 0.145	0.42 ± 0.06	0.49 ± 0.053	1.15 ± 0.185
	Menhinick	1.21 ± 0.165	1.03 ± 0.075	1.1 ± 0.086	1.61 ± 0.273
	Margalef	1.02 ± 0.289	0.79 ± 0.108	0.89 ± 0.098	1.97 ± 0.421
	Equitability_J	1.05 ± 0.011	1 ± 0.069	1.03 ± 0.065	0.93 ± 0.044
	Fisher_alpha	3.16 ± 1.061	2.11 ± 0.42	2.38 ± 0.492	4.75 ± 1.358
	Berger-Parker	0.62 ± 0.087	0.69 ± 0.053	0.62 ± 0.06	0.44 ± 0.072

## Data Availability

The original contributions presented in this study are included in the article/Supplementary Material. Further inquiries can be directed to the corresponding author.

## Supplementary Materials

The following supporting information can be downloaded at https://www.mdpi.com/article/10.3390/insects16040433/s1, Table S1: spatial distribution of mosquito abundance; Table S2: Environmental indices.
