# Supplementary material for: Abundance, Diversity and Distribution of Mosquito Species and Molecular Detection of Its Associated Hepatitis C Virus in Sharkia Governorate, Egypt
Source: Insects. 2025 Apr 19;16(4):433. doi: 10.3390/insects16040433 (PMC12028199; doi:10.3390/insects16040433)
Supplement: Supplementary file 1 [file insects-16-00433-s001.zip › insects-3508908-supplementary.pdf]

Table S1. spatial distribution of mosquito abundance

		**Kafr Saqr**				**Abo Kebeer**				**Diarb Negm**				**El-Zakazik**			
		Hanot	El-Kodah	Abo Shokok	El-Hagarsa	Manshat Radwan	Bane Aiat	Al-Rahmania	Horbat	Safor	El-Asaied	Karmot Sahbara	Saft Zreka	Om El-Zein	Bany Amer	Al-Zankalon	Shanbrt El-Mimona
**Summer**	*C. perexiguus*	8±1.29	9.5±0.85	8.5±1.19	12.5±1.43	8±0.62	8.5±1.25	8.5±1.19	12.5±1.43	15.5±1.19	13±1.29	6.5±1.19	12.5±0.94	6.5±1.22	14.5±0.85	12.5±1.47	14±1.32
	Culex deserticola	4.5±0.64	4.5±0.64	3.5±0.64	6.5±0.85	3.5±0.64	4.5±1.08	3.5±0.64	6.5±0.85	4±0.91	8.5±0.47	2.5±0.47	7±0.62	2±0.86	7.5±1.08	7.5±0.85	8.5±0.85
	Culiseta sp.	5.5±0.64	5.5±1.04	4±0.57	7.5±0.85	5.5±1.25	5±1.49	4±0.57	7.5±0.85	10±1.1	11±0.4	5±0.57	8.5±0.85	6±0.86	10.5±0.85	8.5±0.85	11.5±1.19
	C. antennatus	69.5±3.88	70±4.49	69.5±4.3	74.5±3.01	62.5±2.9	81.5±2.62	69.5±4.3	74.5±3.01	60±2.97	80±2.94	74.5±4.3	76±1.65	62.5±3.88	81±3.3	82.5±4.3	79±4.5
	C. pipiens	109.5±4.66	112.5±3.77	106.5±3.56	120±7.93	121±6.31	124.5±9.25	106.5±3.56	120±7.93	116.5±1.55	129±1.68	127.5±3.77	124.5±2.17	129.5±4.66	114±3.25	129.5±3.56	139±7.61
	C. univittatus	40±3.29	40.5±5.2	40.5±2.49	40.5±3.42	47±3.47	41.5±4.02	40.5±2.49	40.5±3.42	44±2.28	33.5±2.78	43.5±2.49	42.5±2.39	33.5±2.39	40.5±2.52	44.5±2.49	45±3.3
	An. anopheles	26±2.21	26.5±2.62	28±0.91	26±1.49	30±2.44	32±2.5	28±0.91	26±1.49	26±2.38	32.5±1.47	31±0.91	26.5±2.17	26.5±2.62	26.5±1.79	31.5±1.65	30.5±2.13
	An. sergenti	6.5±1.58	6±0.86	7±0.91	7±0.57	9±0.81	7.5±2.32	7±0.91	7±0.57	12±1.93	8.5±1.7	9±0.91	8±0.57	11.5±0.94	9.5±1.03	8±0.95	13±0.57
	An. Multicolor	8.5±2.41	7.5±2.17	8±1.7	11±1.49	11.5±1.04	6.5±1.47	8±1.7	11±1.49	17±1.18	6.5±1.19	12±1.29	12.5±1.25	15.5±0.85	14.5±0.85	11±1.35	15.5±1.19
	An. Coustani	12±0.86	15±0.81	11.5±0.64	12.5±1.04	15±1.25	14±1.49	11.5±0.64	12.5±1.04	18.5±1.37	16±1.49	14.5±0.64	12±0.75	7.5±0.64	18±1.1	9±1.25	10.4
	An. Tenebrosus	8±0.86	8±0.95	8±1.1	7±1.5	6.5±0.7	10±1.41	8±1.1	7±1.5	6±0.4	11.5±1.31	9.5±0.64	7±0.95	11.5±1.04	9±0.62	9.5±1.04	8±0.75
	An. Pharoensis	45.5±3.03	42±2.12	43±4.2	50.5±2.28	41.5±2.66	44.5±4.6	43±4.2	50.5±2.28	44±3.74	49.5±3.81	38±4.2	46±2.19	50.5±2.48	47±2.19	39.5±2.25	42.5±4.66
**Autumn**	*C. perexiguus*	3.5±0.64	5±0.62	3.5±0.47	4.5±0.85	1.5±0.64	4±0.75	3.5±0.47	4.5±0.85	4±0.95	4±1.25	1±0.25	4±0.75	2.5±0.85	3.5±1.08	4.5±0.47	7.5±1.31
	Culex deserticola	0.5±0.47	1.5±0.28	1±0.25	2.5±0.7	1±0.4	2±0.5	1±0.25	2.5±0.7	1±0.57	1±0.62	0.5±0.28	2.5±0.64	0.5±0.28	2±0.75	1±0.4	3±0.4
	Culiseta sp.	1.5±0.47	1.5±0.64	1±0.4	1.5±0.64	1.5±0.47	1.5±0.64	1±0.4	1.5±0.64	2.5±0.7	2±0.25	1.5±0.28	1.5±0.28	2±0.25	1.5±0.28	1.5±0.28	2±0.25
	C. antennatus	17.5±1.95	15±2.21	15.5±2.25	17.5±2.56	16±2.13	15±1.84	15.5±2.25	17.5±2.56	15±0.62	18±1.35	18.5±1.03	19±2.25	14.5±1.95	19±2.21	19.5±1.25	20.5±1.58
	C. pipiens	34.5±5.14	39.5±2.72	38.5±2.05	42±4.87	42±2.78	39±5.26	38.5±2.05	42±4.87	56.5±1.75	51.5±4.52	43.5±2.05	46±3.49	30.5±3.39	46.5±2.56	41±4.13	49±3.54
	C. univittatus	11.5±1.25	13.5±2.17	12±0.75	13±0.81	10±1.29	8±1.1	12±0.75	13±0.81	8±1.49	8.5±1.47	13±1.29	13.5±1.75	8.5±1.65	13±0.91	9.5±0.7	14.5±1.25
	An. anopheles	4±1.68	5±1.32	5.5±1.37	5.5±1.75	5.5±1.95	8±2.28	5.5±1.37	5.5±1.75	5.5±1.25	8.5±1.04	8.5±1.37	7±1.49	6.5±1.08	9±1.65	8.5±0.7	10±1.1
	An. sergenti	1±0.57	2±0.25	1±0.4	0.5±0.47	1.5±0.64	1±0.62	1±0.4	0.5±0.47	2±0.5	2±1.49	1.5±0.47	1.5±0.64	1.5±0.28	2.5±0.85	2.5±0.28	2.5±0.64
	An. Multicolor	0.5±0.47	1.5±0.47	0.5±0.47	0.5±0.28	0.25	1±0.25	0.5±0.47	0.5±0.28	2±0.5	1±0.4	1±0.25	1.5±0.47	1.5±0.28	1.5±0.28	2±0.25	3±0.4
	An. Coustani	4±0.62	2.5±0.28	2.5±0.28	3±0.81	3±0.5	2.5±0.47	2.5±0.28	3±0.81	2±0.25	1.5±0.47	2.5±0.64	3±0.95	2±0.25	2±0.25	2±0.25	2±0.25
	An. Tenebrosus	1±0.57	1±0.4	1±0.4	1±0.57	1±0.25	0.5±0.28	1±0.4	1±0.57	1±0.4	1±0.4	1±0.4	1±0.25	2±0.4	1.5±0.28	1±0.25	1±0.4
	An. Pharoensis	12.5±10.05	15±14.36	12.5±5.08	14±4.57	13±14.88	14.5±1.04	12.5±5.08	14±4.57	14±1.49	13.5±1.47	12.5±0.85	13.5±0.47	20.5±1.75	19±13.36	12±0.62	13.5±0.85
**Winter**	*C. perexiguus*	0	0	0	0	0	0	0	0	0	0	0	0	0	0	0	0
	Culex deserticola	0	0	0	0	0	0	0	0	0	0	0	0	0	0	0	0
	Culiseta sp.	0	0	0	0	0	0	0	0	0	0	0	0	0	0	0	0
	C. antennatus	0.5±0.47	0.25	0.25	0.5	0.25	0	0.25	0.5	0.25	0	0.25	0.5±0.28	0.25	0.25	0	1±0.4
	C. pipiens	0.5±0.7	1.5±0.47	0.25	0.5	1.5±0.28	1.5±0.64	0.25	0.5	1.5±0.28	2.5±0.28	0.5±0.28	1±0.4	1±0.4	2±0.4	1±0.4	1±0.4
	C. univittatus	0	0	0	0	0	0	0	0	0	0	0	0	0	0	0	0
	An. anopheles	0	0.25	0	0	0	0	0	0	0	0	0	0	0	0	0	0
	An. sergenti	0	0	0	0	0	0	0	0	0	0.75	0	0	0	0	0	0
	An. Multicolor	0	0	0	0	0	0	0	0	0	0	0	0	0	0	0	0
	An. Coustani	0	0	0	0	0	0	0	0	0	0	0	0	0	0	0	0
	An. Tenebrosus	0	0	0	0	0	0	0	0	0	0	0	0	0	0	0	0
	An. Pharoensis	0	0	0	0	0	0	0	0	0	0	0	0	0	0	0	0
**Spring**	*C. perexiguus*	0	0.5±0.28	0	0	0	0	0	0	0	0	0	0	0	0	1±0.4	1±0.4
	Culex deserticola	0	0	0	0	0	0	0	0	0	0	0	0	0	0	0	1±0.25
	Culiseta sp.	0	0.5±0.28	0	0	0	0	0	0	0	0	0	0	0	0	1±0.25	1±0.25
	C. antennatus	0.5±0.47	0.25	1±0.25	1.5±0.64	1±0.25	0.5±0.47	1±0.25	1.5±0.64	1±0.25	1±0.25	1±0.25	2±0.25	1.5±0.28	2.5±0.64	2±0.25	3±0.25
	C. pipiens	2.5±0.85	3±0.62	2.5±0.47	2.5±0.94	2±0.25	4.5±1.31	2.5±0.47	2.5±0.94	2±0.25	6±1.88	2.5±0.47	3.5±0.64	7.5±2.17	6.5±1.31	5.5±0.47	6±1.84
	C. univittatus	0	1±0.57	0	0	0	0	0	0	0	0	0	0	0	1.5±0.47	1±0.4	1±0.4
	An. anopheles	0	0.25	0.5±0.28	0	0	0	0	0	0	0	0	0	0	1±0.4	0.5±0.28	1±0.4
	An. sergenti	0	0	0	0	0	0	0	0	0	0	0	0	0	1.5±0.47	1±0.4	0.5±0.28
	An. Multicolor	0	0.5±0.47	0	0	0	0	0	0	0	0	0	0	0	1.5±0.47	1.5±0.28	1.5±0.64
	An. Coustani	0	0	0	0	0	0	0	0	0	0	0	0	0	0.5±0.28	0	0.5±0.28
	An. Tenebrosus	0	0	0	0	0	0	0	0	0	0	0	0	0	0	0	0.25
	An. Pharoensis	0	1±0.25	1±0.4	0.5±0.28	0.5±0.47	0.5±0.28	0	1±0.4	1±0.62	1±0.4	0.5±0.47	0.5±0.47	2.5±0.85	2±0.25	2±0.4	1.5±0.28

Table S2: Environmental indices

		**Kafr Saqr**				**Abo Kebeer**				**Diarb Negm**				**El-Zakazik**			
		Hanot	El-Kodah	Abo Shokok	El-Hagarsa	Manshat Radwan	Bane Aiat	Al-Rahmania	Horbat	Safor	El-Asaied	Karmot Sahbara	Saft Zreka	Om El-Zein	Bany Amer	Al-Zankalon	Shanbrt El-Mimona
**Summer**	Individuals	344.25±5.105	349.75±16.67	336.5±8.808	368.5±6.034	356.25±6.06	377.75±24.448	336.5±8.808	368.5±6.034	374.75±1.493	398.25±10.964	368.5±8.271	382.25±6.835	363.25±4.17	396.25±8.31	393±10.408	415.75±11.7
	Dominance_D	0.17±0.006	0.18±0.004	0.18±0.006	0.17±0.008	0.17±0.007	0.18±0.001	0.18±0.006	0.17±0.008	0.16±0.001	0.17±0.004	0.18±0.006	0.18±0.003	0.19±0.005	0.16±0.004	0.18±0.008	0.18±0.005
	Simpson_1-D	0.82±0.006	0.81±0.004	0.81±0.006	0.82±0.008	0.82±0.007	0.81±0.001	0.81±0.006	0.82±0.008	0.83±0.001	0.82±0.004	0.81±0.006	0.81±0.003	0.8±0.005	0.83±0.004	0.81±0.008	0.81±0.005
	Shannon_H	2±0.028	1.99±0.019	1.98±0.025	2.02±0.026	2.01±0.028	1.96±0.013	1.98±0.025	2.02±0.026	2.09±0.004	2.04±0.019	1.97±0.022	2.01±0.007	1.97±0.011	2.09±0.013	2.01±0.027	2.03±0.015
	Evenness_e^H/S	0.62±0.017	0.61±0.011	0.6±0.015	0.63±0.016	0.62±0.018	0.59±0.007	0.6±0.015	0.63±0.016	0.67±0.003	0.64±0.012	0.59±0.013	0.62±0.004	0.59±0.006	0.67±0.008	0.62±0.017	0.64±0.01
	Brillouin	1.92±0.027	1.91±0.018	1.89±0.025	1.94±0.026	1.92±0.026	1.88±0.014	1.89±0.025	1.94±0.026	2.01±0.004	1.96±0.02	1.89±0.022	1.93±0.008	1.89±0.01	2.01±0.013	1.93±0.029	1.96±0.014
	Menhinick	0.64±0.004	0.64±0.015	0.65±0.008	0.62±0.005	0.63±0.005	0.62±0.02	0.65±0.008	0.62±0.005	0.61±0.001	0.6±0.008	0.62±0.007	0.61±0.005	0.62±0.003	0.6±0.006	0.6±0.008	0.58±0.008
	Margalef	1.88±0.004	1.87±0.015	1.89±0.008	1.86±0.005	1.87±0.005	1.85±0.02	1.89±0.008	1.86±0.005	1.85±0.001	1.83±0.008	1.86±0.006	1.85±0.005	1.86±0.003	1.83±0.006	1.84±0.008	1.82±0.008
	Equitability_J	0.8±0.011	0.8±0.007	0.79±0.01	0.81±0.01	0.8±0.011	0.79±0.005	0.79±0.01	0.81±0.01	0.84±0.001	0.82±0.007	0.79±0.009	0.81±0.002	0.79±0.004	0.84±0.005	0.8±0.011	0.82±0.006
	Fisher_alpha	2.41±0.009	2.41±0.029	2.43±0.015	2.37±0.009	2.39±0.01	2.36±0.037	2.43±0.015	2.37±0.009	2.36±0.002	2.33±0.014	2.37±0.013	2.35±0.01	2.38±0.006	2.33±0.011	2.34±0.014	2.3±0.016
	Berger-Parker	0.31±0.017	0.32±0.007	0.31±0.018	0.31±0.022	0.32±0.014	0.32±0.006	0.31±0.018	0.31±0.022	0.31±0.002	0.32±0.007	0.33±0.015	0.32±0.007	0.35±0.014	0.29±0.012	0.33±0.017	0.33±0.012
**Autumn**	Individuals	100.75±13.616	117±10.785	99±5.115	112±12.013	107.75±14.665	98±4.966	99±5.115	112±12.013	113±4.143	114.5±2.179	105.75±2.926	115.25±6.835	94.25±3.614	134.25±14.15	102±4.242	125.25±4.21
	Dominance_D	0.21±0.017	0.23±0.026	0.22±0.011	0.22±0.004	0.26±0.017	0.22±0.023	0.22±0.011	0.22±0.004	0.28±0.002	0.25±0.027	0.22±0.009	0.22±0.006	0.2±0.012	0.22±0.01	0.2±0.011	0.19±0.008
	Simpson_1-D	0.78±0.017	0.76±0.026	0.77±0.011	0.77±0.004	0.73±0.017	0.77±0.023	0.77±0.011	0.77±0.004	0.71±0.002	0.74±0.027	0.77±0.009	0.77±0.006	0.79±0.012	0.77±0.01	0.79±0.011	0.8±0.008
	Shannon_H	1.79±0.072	1.82±0.082	1.8±0.06	1.8±0.02	1.68±0.065	1.82±0.073	1.8±0.06	1.8±0.02	1.7±0.012	1.76±0.076	1.8±0.032	1.84±0.015	1.89±0.036	1.84±0.043	1.93±0.041	1.96±0.035
	Evenness_e^H/S	0.64±0.047	0.55±0.042	0.58±0.007	0.61±0.045	0.55±0.06	0.59±0.022	0.58±0.007	0.61±0.045	0.51±0.021	0.55±0.035	0.54±0.019	0.57±0.02	0.59±0.017	0.54±0.025	0.58±0.017	0.6±0.023
	Brillouin	1.6±0.053	1.63±0.067	1.6±0.045	1.62±0.006	1.5±0.054	1.62±0.061	1.6±0.045	1.62±0.006	1.51±0.011	1.58±0.069	1.61±0.025	1.65±0.009	1.67±0.027	1.67±0.027	1.71±0.03	1.77±0.032
	Menhinick	0.95±0.072	1.04±0.051	1.05±0.078	0.95±0.06	0.96±0.043	1.06±0.078	1.05±0.078	0.95±0.06	1.01±0.06	1±0.046	1.09±0.056	1.02±0.036	1.16±0.039	1.02±0.055	1.16±0.042	1.05±0.026
	Margalef	1.85±0.134	2.16±0.068	2.07±0.15	1.91±0.133	1.92±0.149	2.07±0.151	2.07±0.15	1.91±0.133	2.06±0.113	2.05±0.102	2.2±0.11	2.1±0.077	2.25±0.064	2.2±0.066	2.32±0.066	2.22±0.051
	Equitability_J	0.8±0.031	0.75±0.032	0.76±0.007	0.78±0.025	0.73±0.041	0.77±0.017	0.76±0.007	0.78±0.025	0.71±0.013	0.74±0.028	0.74±0.012	0.76±0.011	0.78±0.012	0.75±0.018	0.78±0.012	0.79±0.015
	Fisher_alpha	2.6±0.237	3.09±0.137	2.99±0.281	2.67±0.216	2.7±0.23	3±0.275	2.99±0.281	2.67±0.216	2.93±0.211	2.91±0.179	3.19±0.206	3±0.134	3.34±0.133	3.12±0.14	3.44±0.136	3.18±0.094
	Berger-Parker	0.35±0.023	0.39±0.03	0.38±0.019	0.39±0.007	0.44±0.017	0.4±0.042	0.38±0.019	0.39±0.007	0.49±0.002	0.45±0.034	0.4±0.012	0.4±0.012	0.34±0.021	0.38±0.009	0.37±0.027	0.37±0.017
**Winter**	Individuals	2.33±0.881	2.33±0.881	1±0	2±0	1.75±0.25	2±0.577	1±0	2±0	1.75±0.25	3.25±0.629	1±0	1.5±0.288	1.66±0.333	2.25±0.629	1.33±0.333	2±0.577
	Dominance_D	0.75±0.25	0.58±0.416	---	1±0	0.66±0.333	1±0	---	1±0	0.66±0.333	0.85±0.15		0.5±0.5	0.5±0.5	0.83±0.166	---	0.33±0
	Simpson_1-D	0.25±0.25	0.41±0.416	---	0	0.33±0.333	0	---	0	0.33±0.333	0.15±0.15		0.5±0.5	0.5±0.5	0.16±0.166	---	0.66±0
	Shannon_H	0.22±0.229	0.43±0.43	0	0	0.23±0.235	0	0	0	0.23±0.235	0.19±0.193	0	0.23±0.235	0.31±0.314	0.17±0.171	0	0.4±0.231
	Evenness_e^H/S	0.99±0.001	1.07±0.07	1±0	1±0	1.07±0.071	1±0	1±0	1±0	1.07±0.071	1.02±0.02	1±0	1.07±0.071	1.09±0.094	0.99±0.001	1±0	1.05±0.033
	Brillouin	0.11±0.115	0.2±0.207	0	0	0.08±0.086	0	0	0	0.08±0.086	0.11±0.115	0	0.08±0.086	0.11±0.115	0.08±0.086	0	0.18±0.105
	Menhinick	0.9±0.097	1.06±0.231	1±0	0.7±0	0.95±0.167	0.76±0.124	1±0	0.7±0	0.95±0.167	0.68±0.074	1±0	1.03±0.145	1.04±0.205	0.85±0.084	0.9±0.097	1.07±0.044
	Margalef	0.24±0.24	0.48±0.481	0	0	0.36±0.36	0	0	0	0.36±0.36	0.15±0.155	0	0.36±0.36	0.48±0.481	0.18±0.18	0	0.45±0.262
	Equitability_J	---	---	---	---	---	---	---	---	---	---	---	---	---	---	---	1.15±0
	Fisher_alpha	0.79±0.459	2.08±1.7	0	0.79±0	0.39±0.229	0.44±0.233	0	0.79±0	0.39±0.229	0.77±0.167	0	0.19±0.198	0.26±0.265	0.79±0.324	0.26±0.265	1.31±0.756
	Berger-Parker	0.91±0.083	0.83±0.166	1±0	1±0	0.87±0.125	1±0	1±0	1±0	0.87±0.125	0.9±0.099	1±0	0.87±0.125	0.83±0.166	0.93±0.062	1±0	0.83±0.096
**Spring**	Individuals	3.5±1.258	7.75±1.25	5±0.408	5.25±1.108	4.25±0.946	6.66±0.333	3.5±0.645	5.75±1.376	4.75±1.108	8±2.121	3.75±1.108	6.5±0.866	13±2.041	17.75±2.78	15.25±0.478	18.5±2.217
	Domice_D	0.61±0.198	0.21±0.09	0.28±0.064	0.41±0.07	0.26±0.04	0.57±0.119	0.6±0.141	0.46±0.188	0.22±0.039	0.59±0.175	0.3±0.019	0.36±0.057	0.49±0.073	0.2±0.034	0.15±0.024	0.15±0.042
	Simpson_1-D	0.38±0.198	0.78±0.09	0.71±0.064	0.58±0.07	0.73±0.04	0.42±0.119	0.39±0.141	0.53±0.188	0.77±0.039	0.4±0.175	0.7±0.019	0.63±0.057	0.5±0.073	0.79±0.034	0.84±0.024	0.84±0.042
	Shannon_H	0.36±0.214	1.54±0.242	1.15±0.172	0.82±0.123	1.02±0.131	0.7±0.197	0.52±0.179	0.86±0.297	1.14±0.116	0.7±0.271	0.78±0.274	0.94±0.123	0.88±0.089	1.77±0.159	1.99±0.12	2.11±0.169
	Evenness_e^H/S	1.02±0.031	1.11±0.091	1.1±0.071	1.03±0.041	1.14±0.022	0.88±0.057	1±0.042	1.05±0.074	1.16±0.027	0.91±0.126	1.06±0.024	1.05±0.039	0.81±0.071	0.91±0.048	0.99±0.033	0.96±0.093
	Brillouin	0.2±0.116	0.89±0.158	0.6±0.077	0.45±0.077	0.52±0.098	0.41±0.125	0.25±0.086	0.49±0.17	0.59±0.087	0.39±0.142	0.41±0.152	0.58±0.086	0.61±0.072	1.24±0.135	1.32±0.061	1.43±0.108
	Menhinick	0.87±0.128	1.61±0.213	1.33±0.165	1.02±0.114	1.23±0.088	0.9±0.117	0.93±0.094	1.05±0.19	1.3±0.114	0.93±0.217	1.18±0.071	0.98±0.081	0.85±0.057	1.59±0.113	1.92±0.184	2.08±0.22
	Margalef	0.35±0.22	1.7±0.314	1.23±0.236	0.8±0.135	1.07±0.126	0.7±0.164	0.56±0.197	0.84±0.304	1.18±0.146	0.77±0.296	0.81±0.28	0.8±0.117	0.79±0.041	1.98±0.234	2.39±0.254	2.73±0.365
	Equitability_J	1.06±0.096	1.05±0.069	1.08±0.057	1.03±0.055	1.15±0.013	0.82±0.091	1±0.084	1.05±0.091	1.15±0.017	0.85±0.178	1.09±0.033	1.06±0.048	0.8±0.081	0.94±0.026	0.99±0.018	0.97±0.049
	Fisher_alpha	1.02±0.567	5.68±1.817	4.1±1.755	1.86±0.402	3.17±0.774	1.32±0.333	1.56±0.389	2.38±1.067	3.81±0.961	2.12±1.132	2.04±0.7	1.56±0.225	1.27±0.097	3.99±0.531	6.42±1.582	7.33±1.628
	Berger-Parker	0.84±0.089	0.44±0.095	0.54±0.058	0.66±0.058	0.56±0.06	0.75±0.091	0.8±0.07	0.64±0.127	0.51±0.059	0.76±0.106	0.69±0.108	0.54±0.07	0.65±0.083	0.41±0.042	0.37±0.032	0.33±0.068
